# The effect of ding’s screw and tension band wiring for treatment of olecranon fractures: a finite element study

**DOI:** 10.1186/s12891-023-06684-4

**Published:** 2023-07-24

**Authors:** Nuo Yin, Mingmang Pan, Chenglei Li, Li Du, Liang Ding

**Affiliations:** Department of Orthopaedics, Shanghai Jiao Tong University Affiliated Sixth People’s Hospital South Campus, Shanghai, 201400 China

**Keywords:** Olecranon fractures, Tension band wiring, Ding’s screw and tension band wiring, Finite element, Complications

## Abstract

**Background:**

Tension band wiring (TBW) is a common surgical intervention for olecranon fractures. However, high rate of complications such as loss of reduction, skin irritation, and migration of the K-wires were reported up to 80%. Ding’s screw tension band wiring (DSTBW) is a new TBW technique that has shown positive results in the treatment of other fracture types. The objective of this study was to evaluate the stability of DSTBW in the treatment of olecranon fractures by finite element analysis.

**Method:**

We used Ding’s screw tension band fixation (DSTBW) and K-wire tension band fixation (TBW) to establish a finite element model to simulate and fix olecranon fractures. The stress distribution, opening angle, twisting angle, and pullout strength of K-wires or screws were analyzed and compared.

**Results:**

The maximum von Mises stress was observed on the internal fixation for 90° elbow motion in both groups. The von Mises value of the screw in DSTBW was 241.2 MPa, and the von Mises value of k-wire in TBW was 405.0 MPa. Opening angle: TBW was 0.730° and DSTBW was 0.741° at 45° flexion; TBW was 0.679° and DSTBW was 0.693° at 90° flexion. Twisting angle: TBW was 0.146° and DSTBW was 0.180° at 45° flexion; TBW was 0.111° and DSTBW was 0.134° at 90° flexion. The pullout strength of DSTBW was significantly higher than that of TBW. Maximum pullout strength of Ding’s screw was 2179.1 N, maximum pullout strength of K-wire was 263.6 N.

**Conclusion:**

DSTBW technology provides stable fixation for olecranon fractures, reducing the risk of internal fixation migration and failure.

**Supplementary Information:**

The online version contains supplementary material available at 10.1186/s12891-023-06684-4.

## Background

Olecranon fractures are relatively common fractures, accounting for about 10% of adult upper limb fractures, and are more frequent in young patients after high-energy trauma or the elderly after low-energy fall [1]. Olecranon fractures are usually associated with the articular surface and require surgical treatment [2]. The goals of surgical treatment are to reduce the fracture and restore joint stability, allowing for early functional exercise. There are a series of surgical interventions for olecranon fractures, including tension band wiring (TBW) [3], plate fixation [4], and intramedullary screws (IM) [5]. TBW is a simple, low-cost technique that relies on the principle of converting posterior tension into joint compression force and is the most widely used technique for displaced, non-comminuted olecranon fractures [6–8].

However, the incidence of complications associated with TBW is high (up to 80%), such as loss of reduction, olecranon bursitis, Kirschner wire (K-wire) migration, and skin irritation are often reported [9–11]. The subcutaneous nature and potential displacement of K-wires may be the cause of local pain and discomfort in patients. However, removing metalware does not always solve these symptoms. More than 65% of patients still experienced mild pain or discomfort after the metalware was removed after TBW [6]. In long term, whether the metalware is removed or not, the low level of pain is noticeable and degenerative changes have been developed [6].

**Fig. 1:**
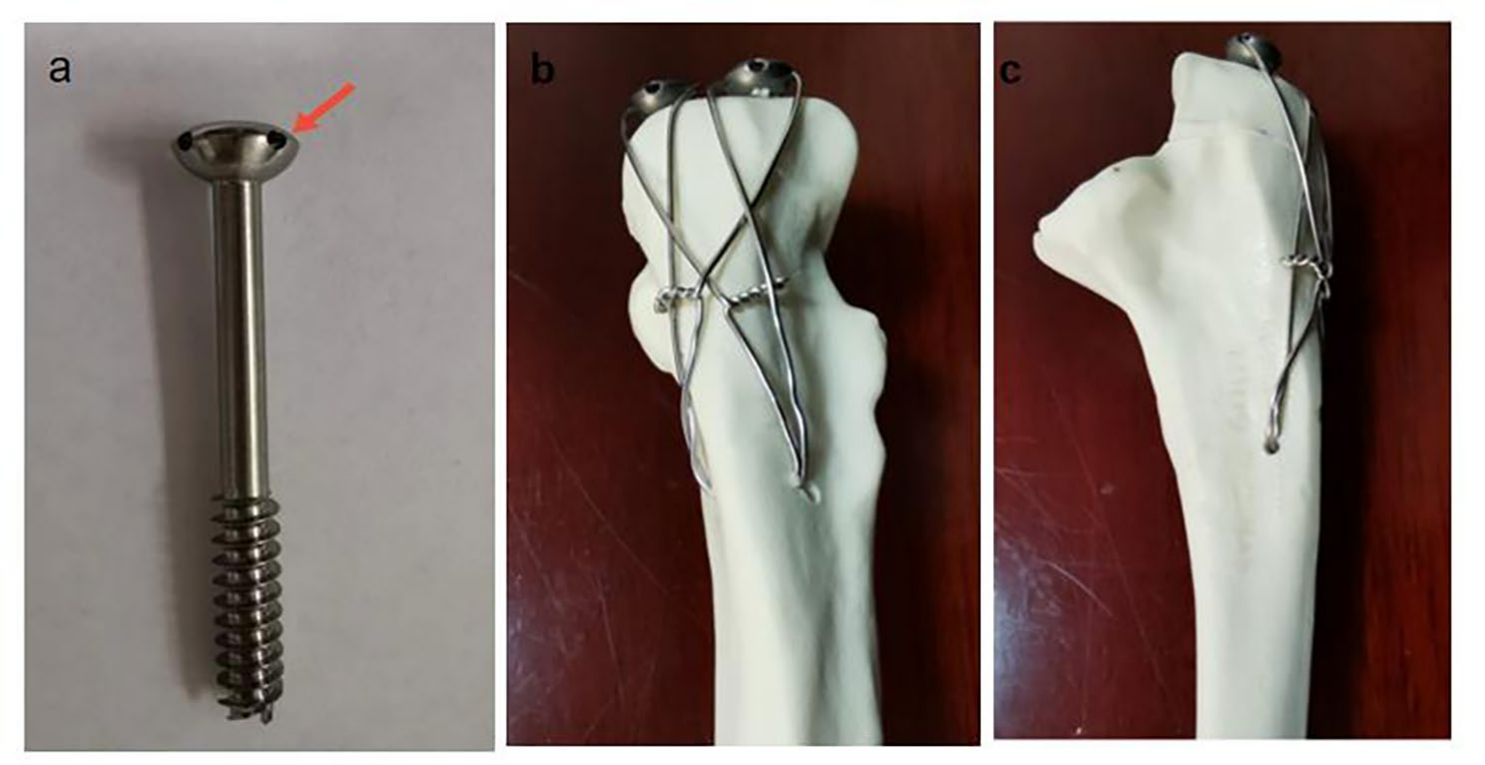
The appearance of Ding’s screw(a) and red arrow showing the hole at the tail. Anterior (b) and lateral (c) view of a sawbone model show the DSTBW fixation technique

DSTBW is a new TBW technique that has shown positive results in the treatment of inferior pole patellar fractures [12]. The using of DSTBW technology(Fig. 1)in olecranon fractures may overcome these problems caused by K-wires in conventional TBW. In theory, the steel wires pass through the holes at the end of the Ding’ s screw, creating a solid " integrated structure” that makes the screws difficult to migrate. At the same time, the end of the screw is much smaller than the end of the curved K-wire, which can effectively reduce skin irritation symptoms. The objective of this study was to evaluate the effect of DSTBW on olecranon fracture by finite element analysis.

## Methods

### Finite element Biomechanical Study

#### Collection of imaging data

Radiologic images of a normal olecranon from a 28-year-old male were obtained from 0.5-mm width cuts of 64-slice computed tomography scans to observe bone tissue. The scanning conditions were as follows: 155 mA at 120 kV. The scanned CT data was saved in 512 × 512 pixels DICOM format.

#### Finite element model of proximal ulna construction

Radiological images in DICOM format were imported into Mimics 17 (Materialise, Belgium) to develop a 3D(three-dimensional) model of proximal ulna. Threshold segmentation, region growth, and calculation of the 3D model were performed in Mimics 17 software, and physicochemical processing was performed in software Imageware 13.0 (Siemens, Plano, TX) and Geomagic 2012 (Cary, NC) to build the olecranon model.

**Fig. 2:**
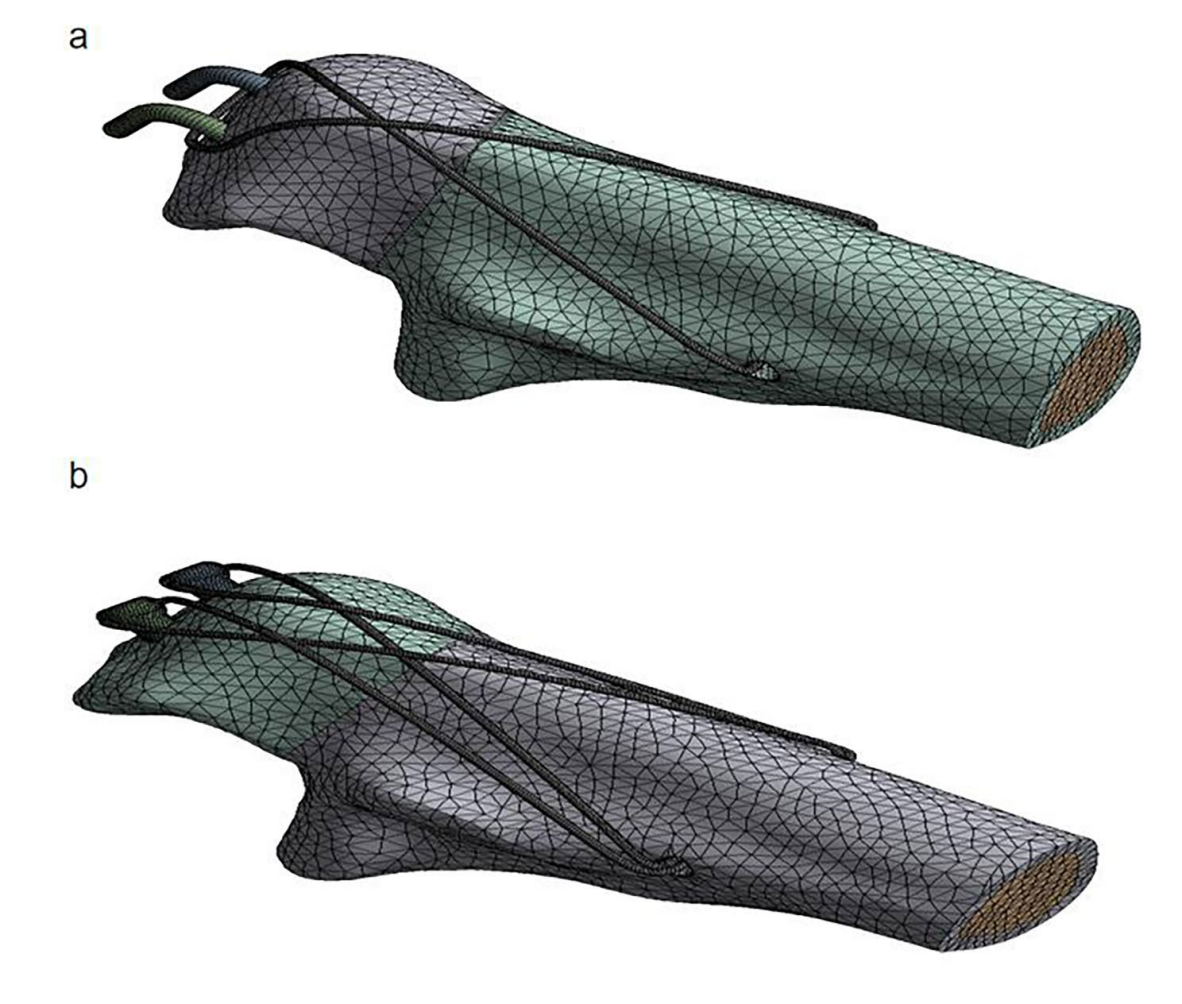
Meshing of the TBW(a) and DSTBW(b) model

The Mayo type IIA type olecranon fracture line [10] was created and two different surgical procedures were performed for the fixation of the fracture by ProE 5.0 software (PTC Inc., Boston, MA). In the TBW group, the fracture was fixed by 2 bicortically placed K-wires. A 2.0-mm hole was made perpendicular to the ulnar shaft, an 18-gauge metal wire was then used to make a figure-of-eight wiring between the hole and the K-wires (Fig. 2a). In the DSTBW group, the fracture was fixed by two Ding’s screws (Double Medical Technology Inc, Xiamen, Fujian, China) which inserted from the tip of the olecranon through the proximal ulna, passing as close as possible to the subchondral bone. A 2.0-mm hole was made perpendicular to the ulnar shaft. Two 18-gauge metal wire were passed through the holes on the tail end of Ding’s screws separately and used to make the figure-of-eight wiring between the hole and Ding’s screws (Fig. 2b).

#### Volume mesh generation

The model of the established combined with the internal fixation system was output using step format and imported into the ANSYS Workbench 2020R2 software (ANSYS, Inc., Pittsburgh, PA, USA). The FE meshes were generated as a tetrahedral 1.6 mm for olecranon and 0.3 mm for K-wire, screw and tension band. The average mesh quality was 0.82. The Ding’s screw was modeled as 4 mm thick and 50 mm long. The K-wire was modeled as 2 mm thick, and the steel wire was 1.25 mm thick. In this study, there were 127,808 elements with 285,000 nodes in TBW model after meshing, and 135,642 elements with 302,470 nodes in DSTBW model.

#### Assignment of material properties

According to previous literatures [13–15], the material parameters were set (Table 1). The Poisson’s ratio was set to 0.3 for cortical bone and cancellous bone, and the elastic modulus were set to 18 GPa and 5 GPa respectively. The Ding’s screw and K-wires were modeled as titanium alloy Ti6Al4V. The material constants were as follows: the elastic modulus 106 Gpa and Poisson’s ratio 0.33. In the case of the steel wire, the elastic modulus and Poisson ratio were set to 210 GPa and 0.3, respectively.We change the “Tolerance Type” under the connections option in the software to “Value” and set the “Tolerance Value” to 0.05. The connection was automatically created by the software. According to previous literatures [16, 17], the contact definition was set: The relationship between cortical bone and cancellous bone was set as Bonded, the bone pieces at both ends of the fracture line were set as Rough, and the other relationships were set as No Separation.

**Table 1:** Material parameters for the bone and fixation systems

Component name	Young’s modulus (GPa)	Poisson’s ratio
Cortical bone	18	0.3
Cancellous bone	5	0.3
K-wire	210	0.3
steel wire	210	0.3
Ding’s screw	106	0.33

#### Loading and boundary conditions

**Fig. 3:**
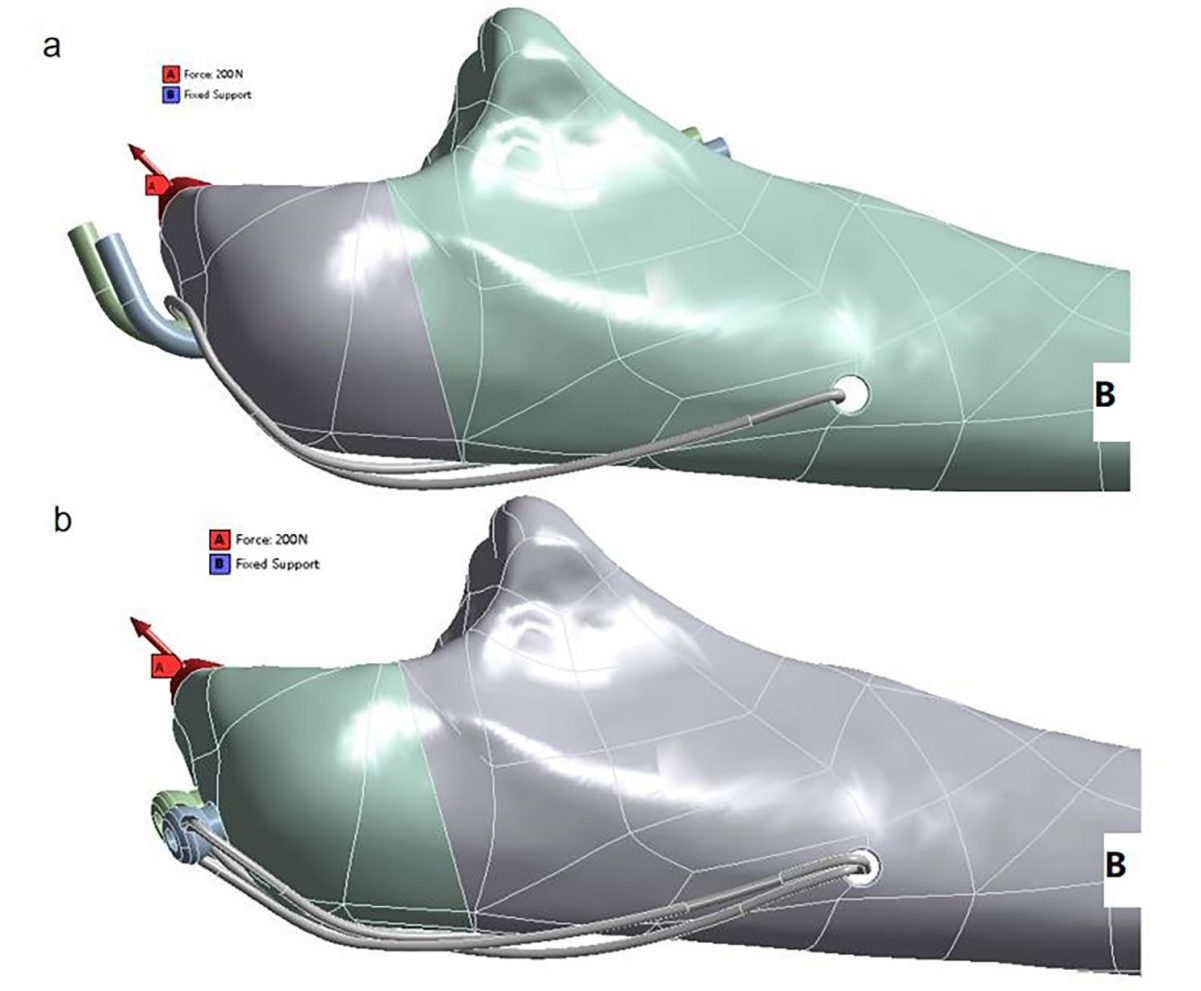
Loading and boundary conditions in DSTBW(a) and TBW(b).

A mesh was generated with an element size from 4 mm to 0.3 mm (supplementary material 1) and a convergence analysis with 5% tolerance [18] was selected. The distal ulna point B (Fig. 3) was determined by ANSYS program, and the pulling direction of the triceps muscle to the proximal olecranon projection was simulated. A tensile force of 120 N for 90° and 200 N for 45° was applied according to previous literatures [13, 19].

After the mechanical loads were defined, the distribution of von Mises stress on fixation and the changes of opening and twisting angles in the fracture line were evaluated. Applying an axial displacement loading, record and draw the loading and displacement curves of K-wires or screws. The peak is considered as the maximum pullout strength.

## Results

### Finite element analysis: von mises stress distribution

**Fig. 4:**
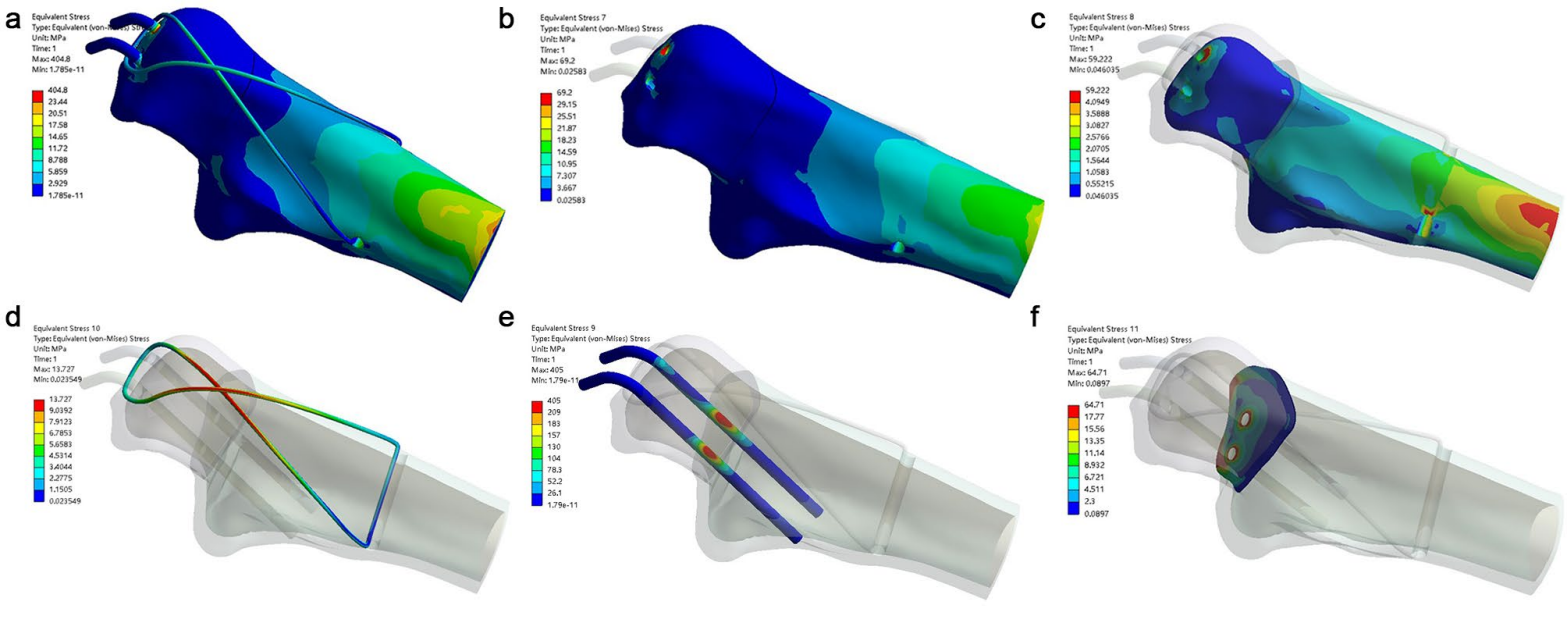
Von Mises stress distribution of the internal fixator at 90° elbow movement in the TBW group. (a, stress distribution on TBW; b, cortical bone; c, cancellous bone; d, steel wire; e, K-wire; f, fracture site)

**Fig. 5:**
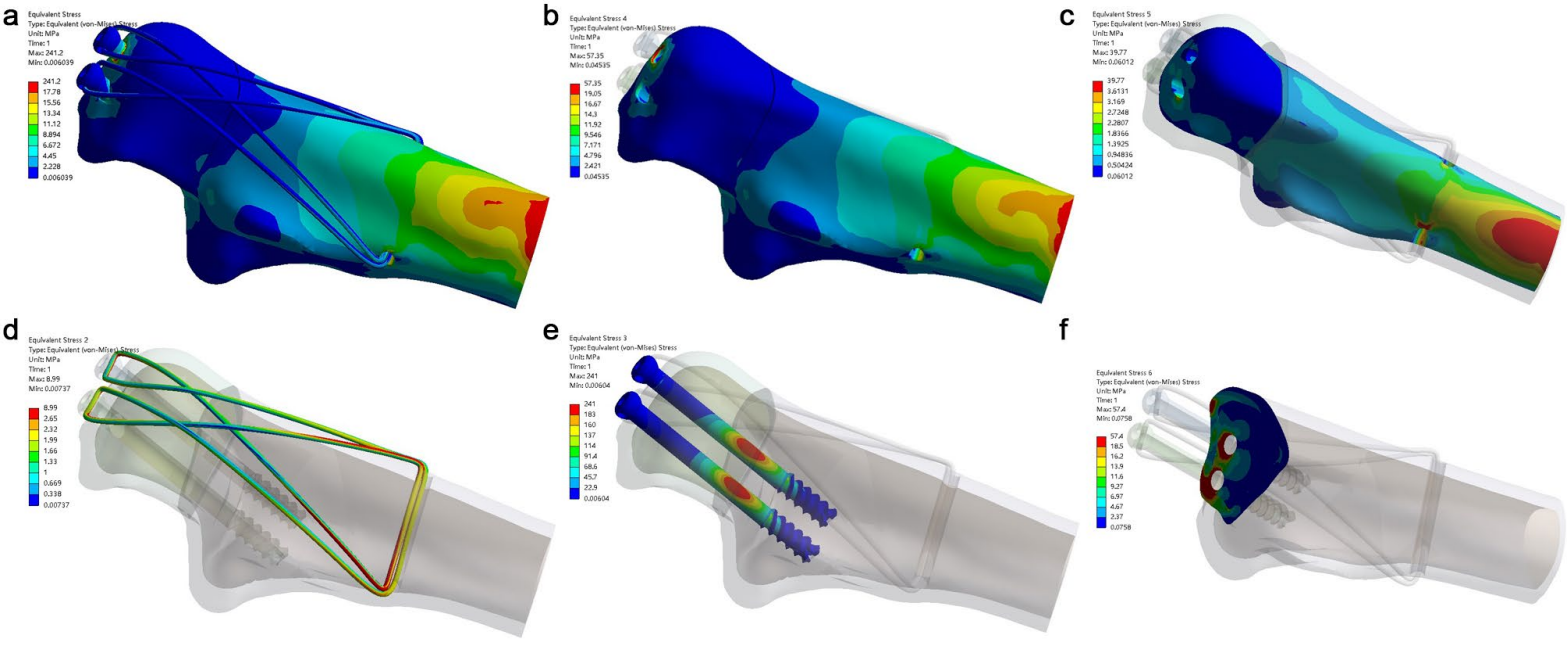
Von Mises stress distribution of the internal fixator at 90° elbow movement in the DSTBW group. (a, stress distribution on DSTBW; b, cortical bone; c, cancellous bone; d, steel wire; e, Ding’s screw; f, fracture site)

There was no fixation failure in either fixation system. Compared with TBW, DSTBW has smaller von Mises stress distribution at different elbow movements (45° and 90°) (Table 2). The maximum von Mises stress in DSTBW was 212.0 MPa and 241.2 MPa at 45° and 90° elbow movements, respectively. The maximum von Mises stress at k-wire was 366.5 MPa and 405.0 MPa at 45° and 90° elbow movements, respectively (Figs. 4 and 5). The stress distribution of the internal fixation during 45° elbow movement in the TBW and DSTBW groups is shown in the supplementary materials 2.

**Table 2:** Von Mises stress (MPa) in different elbow movement

Component name	Von Mises stress (MPa)			
	**DSTBW**		**TBW**	
	45°	90°	45°	90°
**Cortical bone**	56.6	57.4	69.4	69.2
**Cancellous bone**	39.8	54	49.3	59.2
** K-wire**	‒	‒	366.5	405.0
**steel wire**	22.3	9.0	7.2	13.7
**Ding’s screw**	212.0	241	‒	‒
**Fracture site**	56.6	57.4	67.2	64.7

### Finite element analysis: opening and twisting angle

For TBW method; the opening angle was recorded 0.730°,0.679° at 45° and 90° elbow flexion position respectively (Fig. 5). For DSTBW method; the opening angle was recorded 0.741°, 0.693° at 45° and 90° elbow flexion position respectively.

For TBW group, the twisting angle was recorded 0.146°, 0.111° at 45° and 90° elbow flexion position respectively. For DSTBW group, the twisting angle was recorded 0.180°, 0.134° at 45° and 90° elbow flexion position respectively. The opening angle and twisting angle of TBW and DSTBW were similar in 45° and 90° elbow flexion position.

### Finite element analysis: pullout strength

**Fig. 6:**
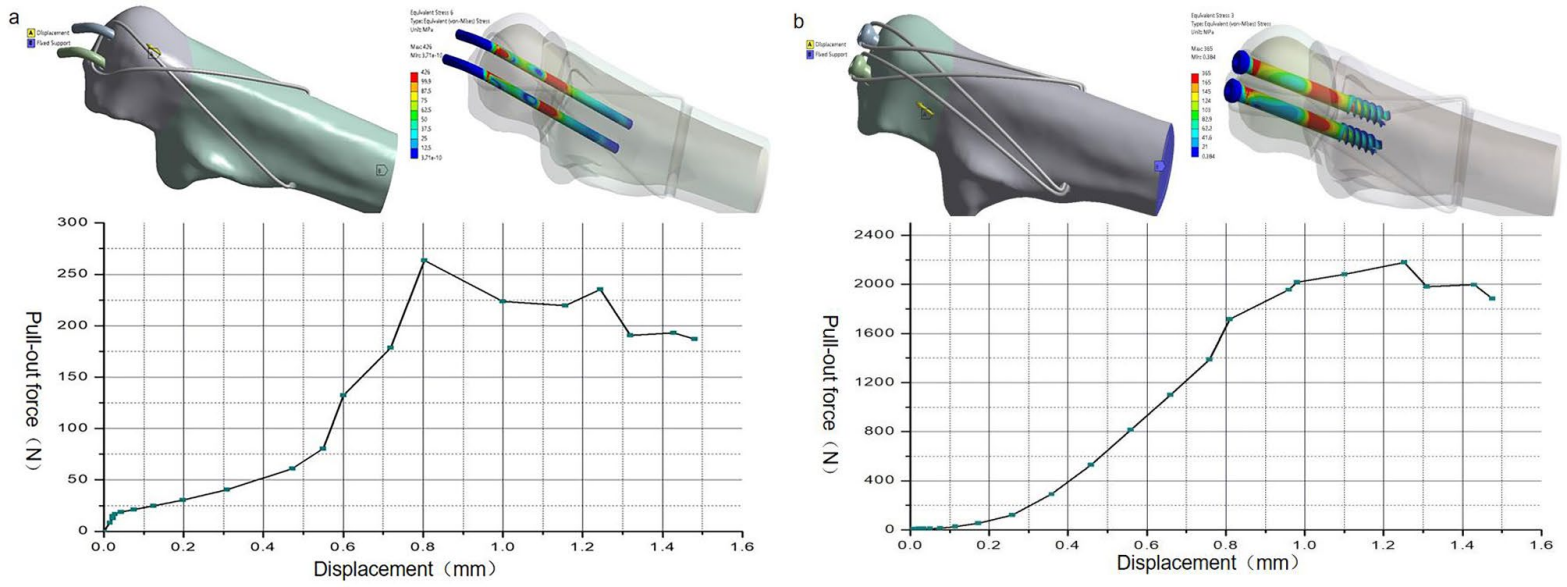
The pullout strength of K-wires in TBW model(a) and screws in DSTBW model(b). After axial displacement loading is applied to the model, the load and displacement of the K-wires or screws are recorded, and the curve is drawn. The peak is the maximum pullout strength

The pullout strength of DSTBW was significantly higher than that of TBW(P < 0.001). The average maximum pullout strength for the Ding’s screws was 2179.1 N. The average maximum pullout strength for the K-wires was 263.6 N (Fig. 6). The results indicated that the Ding’s screws increased stability and reduced the risk of migration of medical apparatus and instruments.

## Discussion

TBW has been generally accepted as the standard treatment for Mayo type IIA olecranon fractures [3, 13]. Although this technique has shown excellent outcomes in both biomechanical and clinical studies [20–23], it has been reported that the incidence of complications and reoperation is very high [9–11]. Patient’s local pain and discomfort may be caused by the excessive tail of the curved K-wire and its potential migration. Therefore, improved techniques are used to improve the strength of fixation and reduce complications, such as cannulated screws. Cannulated screws have been reported for the treatment of olecranon fractures, including IM and IM-TBW [24]. However, previous studies have shown that fixation using IM screws is technically challenging and unreliable [25–27]. The IM-TBW technique provides better structural stability than fixation with the IM screw alone [28, 29]. Nevertheless, this technique requires the use of a large washer combined with steel wire to form tension bands, and the use of washers has a higher complication rate than using screws alone [30]. This may be the reason why this technique is rarely used by orthopedic surgeons. Edwards et al. [31] observed that IM-TBW technique was used in about 6% of cases in their multicenter study.

Therefore, in view of the shortcomings of the above fixations, the Ding’s screws have the following advantages: First, DSTBW is connected by steel wires through holes at the end of the Ding’s screw to form a strong “integral structure”, which effectively increases mechanical stability. At the same time, the end of the Ding’s screws is much smaller than the end of the curved k-wire and large washers, which can effectively avoid the k-wire migration and skin irritation symptoms. Second, compared with IM-TBW, there is no need to use large washers, which reduces complications caused by washers. Third, Ding’s screw has four holes at the end, which is designed to facilitate operation and simplify the procedure of steel wire passing through the holes at the end. When the steel wire is crossed, the remaining holes allow additional ultrabraid sutures to pass through, which is helpful to fix small fragments of fracture. If necessary, the ultrabraid sutures can also be sutured to the triceps tendon to further enhance the stability of internal fixation.

In this study, a finite element study was conducted on the stability of DSTBW in the treatment of olecranon fracture to provide a basis for its clinical application. The stress distribution of cortical bone, cancellous bone, K-wire, steel wire and Ding’s screws was tested after the model was constrained and loaded. The results of the finite element model show that the von Mises stress on DSTBW is smaller than that on TBW when the elbow joint is moving, indicating that DSTBW is less likely to be failed. Compared with TBW, the pullout strength of DSTBW is much higher after applying an axial load. When these findings are applied to clinical practice, it may be postulated that DSTBW will provide sufficient stability to reduce the risk of internal fixation migration and failure.

This study has some limitations. It is difficult to analysis the soft tissue structures in a finite element model, especially elbow is a complex joint with synovial fluid, multiple muscles and ligaments. The elbow joint is a hinge joint formed by the meeting of the humerus, radius and ulna. The biomechanical effects of humerus and radius were also ignored in this study. Further biomechanical experiments include all soft tissues, bony structures and the natural elbow movements are needed to verify the results.

## Conclusion

In conclusion, the finite element analysis suggests that the DSTBW technique can provide stable fixation for olecranon fractures, reducing the risk of internal fixation migration and failure.

## Electronic supplementary material

Below is the link to the electronic supplementary material.

Supplementary materials 1

Supplementary material 2

## Data Availability

All data generated or analyzed during this study are included in this published article [and its supplementary information files].

## Abbreviations

3D: Three-dimensional
K-wire: Kirschner wire
IM: Intramedullary screw
TBW: Tension band wiring
DSTBW: Ding’s screw and tension band wiring
