# Supplementary material for: The effect of ding’s screw and tension band wiring for treatment of olecranon fractures: a finite element study
Source: BMC Musculoskelet Disord. 2023 Jul 24;24:603. doi: 10.1186/s12891-023-06684-4 (PMC10364372; doi:10.1186/s12891-023-06684-4)
Supplement: Supplementary file 1 — Supplementary materials 1 [file 12891_2023_6684_MOESM1_ESM.docx]

**TABLE 1**   **Mesh convergence analyses of TBW model.**

| Element size/mm | nodes | elements | maximum von Mises stresses /Mpa | change/% |
| --- | --- | --- | --- | --- |
| 4 | 66181 | 35999 | 223.9 | - |
| 2 | 67752 | 36630 | 274 | 22.4 |
| 1 | 74275 | 40306 | 325.8 | 18.9 |
| 0.5 | 133734 | 67076 | 362.6 | 11.3 |
| 0.4 | 170087 | 83822 | 390.2 | 7.6 |
| 0.3 | 285000 | 127808 | 405 | 3.8 |
