# Supplementary figures and images for: The effect of ding’s screw and tension band wiring for treatment of olecranon fractures: a finite element study
Source: BMC Musculoskelet Disord. 2023 Jul 24;24:603. doi: 10.1186/s12891-023-06684-4 (PMC10364372; doi:10.1186/s12891-023-06684-4)

Supplementary materials 2_45°elbow movement.zip

Fig. a
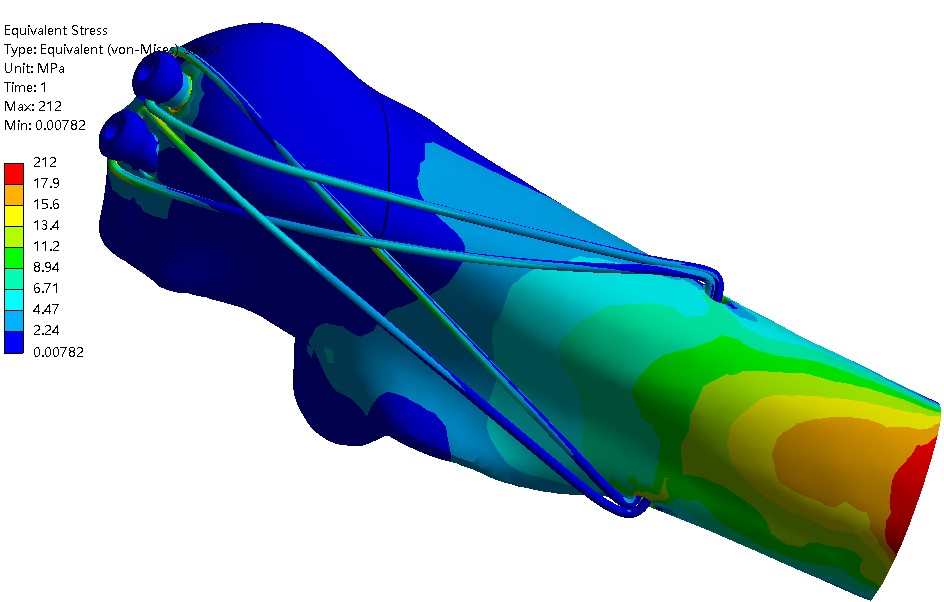


Fig. b
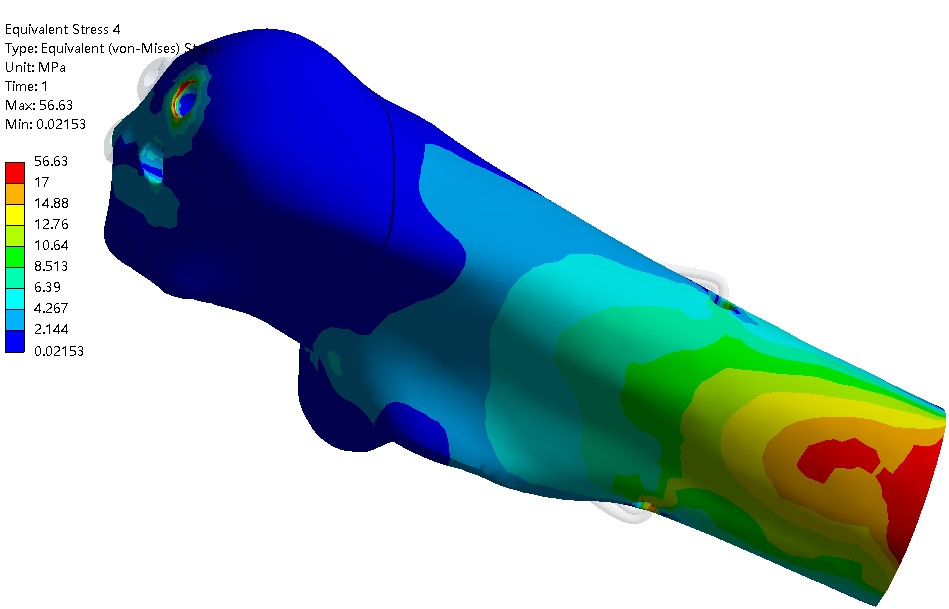


Fig. c
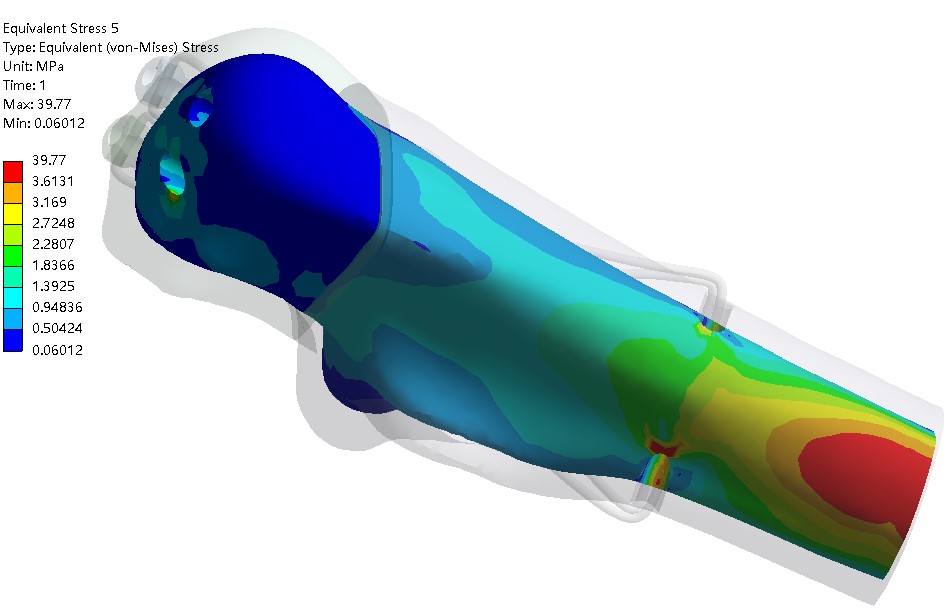


Fig. d
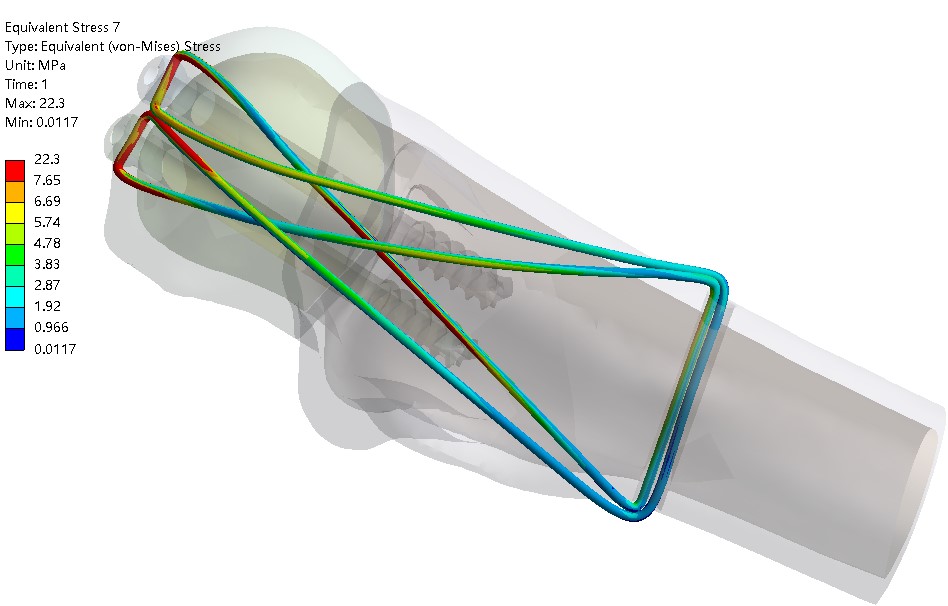


Fig. e
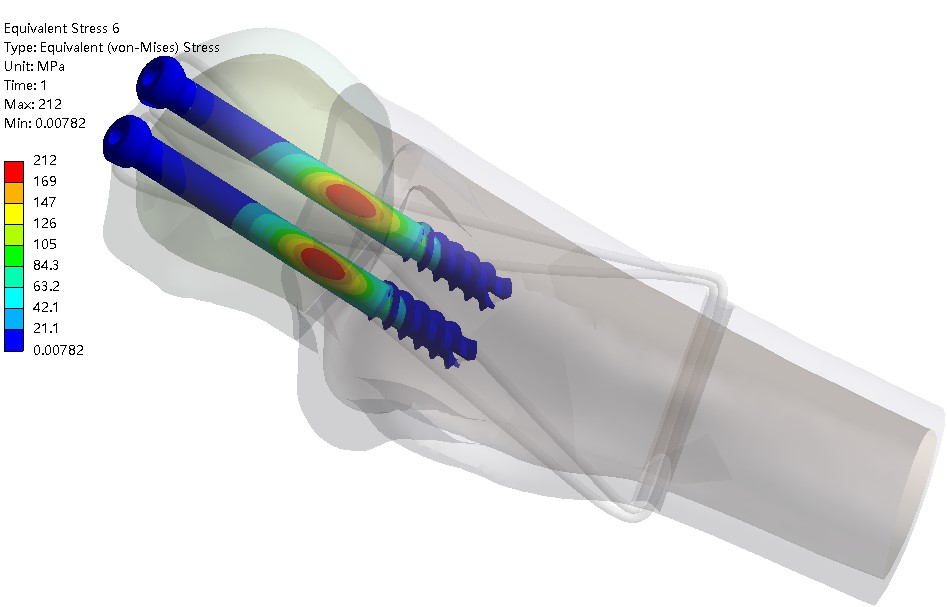


Fig. f
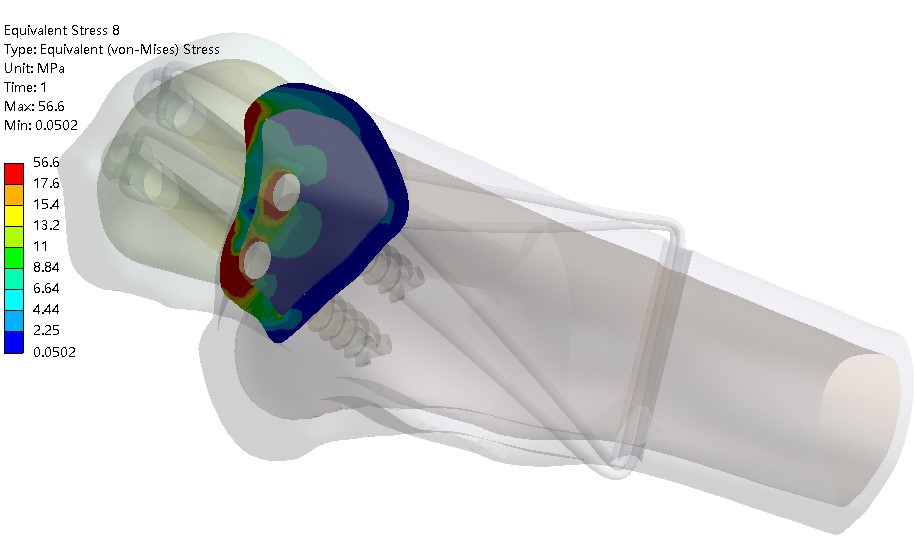

Supplement: Supplementary file 2 — Supplementary material 2 [file 12891_2023_6684_MOESM2_ESM.docx]
